# Eyebrow movements as signals of communicative problems in human face-to-face interaction

**DOI:** 10.1098/rsos.241632

**Published:** 2025-03-12

**Authors:** Paul Hömke, Stephen C. Levinson, Alexandra K. Emmendorfer, Judith Holler

**Affiliations:** ^1^ Max Planck Institute for Psycholinguistics, Nijmegen, The Netherlands; ^2^ Donders Institute for Brain, Cognition and Behaviour, Radboud University Nijmegen, Nijmegen, The Netherlands

**Keywords:** repair, eyebrow movements, raises, furrows, conversation, facial signals

## Abstract

Repair is a core building block of human communication, allowing us to address problems of understanding in conversation. Past research has uncovered the basic mechanisms by which interactants signal and solve such problems. However, the focus has been on verbal interaction, neglecting the fact that human communication is inherently multimodal. Here, we focus on a visual signal particularly prevalent in signalling problems of understanding: eyebrow furrows and raises. We present, first, a corpus study showing that differences in eyebrow actions (furrows versus raises) were systematically associated with differences in the format of verbal repair initiations. Second, we present a follow-up study using an avatar that allowed us to test the causal consequences of addressee eyebrow movements, zooming into the effect of eyebrow furrows as signals of trouble in understanding in particular. The results revealed that addressees’ eyebrow furrows have a striking effect on speakers’ speech, leading speakers to produce answers to questions several seconds longer than when not perceiving addressee eyebrow furrows while speaking. Together, the findings demonstrate that eyebrow movements play a communicative role in initiating repair during conversation rather than being merely epiphenomenal and that their occurrence can critically influence linguistic behaviour. Thus, eyebrow movements should be considered core coordination devices in human conversational interaction.

## 1. Introduction

Unlike most other animals, humans tend to face each other in everyday communication. This allows them to rely not only on vocal but also on visual bodily behaviours when communicating [1]. While the language sciences have made substantial progress in the study of hand gestures (e.g. [2–6]), the face has still received comparatively little attention.

It is well known that the face can play an important role in expressing emotions [7,8]. *Facial expressions* are often considered to be involuntary manifestations of an individual’s emotion (e.g. fear upon seeing a spider), and they have been distinguished from more voluntary *facial gestures* ([5,9,10]; see also ‘conversational facial signals’ and ‘facial displays’, [11,12]). Facial gestures are considered to form part of a social-interactive process and the structure and content of conversation, rather than expressions of an individual’s inner emotions [13,14]. As such, they fulfil the function of communicative signals. They can also serve as depictions [15], such as when impersonating a particular character during story telling (see also [16–18]), and of course, gaze direction has long been acknowledged to play a fundamental role in signalling communicative intentions (e.g. [19,20]).

**Figure 1.**
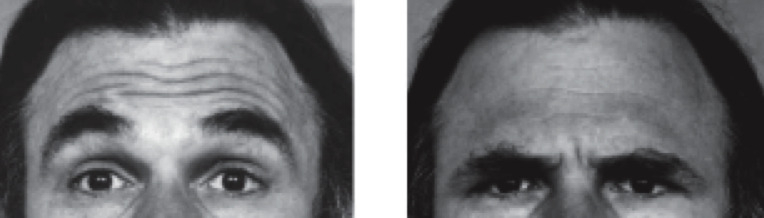
Example images of an eyebrow raise (AU1 + 2) and an eyebrow furrow (AU4) (source: [22], copyright obtained by P.H.).

Some of the most prevalent facial movements in conversation are eyebrow raises and furrows. According to the Facial Action Coding System [21,22]—a system that allows for coding visually distinguishable facial movements (termed action units (AU))—eyebrow raises are realized by the inner brow raiser (central frontalis; AU1) together with the outer brow raiser (lateral frontalis; AU2), while eyebrow furrows are realized by the brow lowerer (corrugator, depressor supercilii, depressor glabellae; AU4; see figure 1).

In the emotion domain, eyebrow raises have been associated with positive emotions like surprise, and eyebrow furrows with negative emotions like anger [8]. In terms of non-emotional signalling, eyebrow movements have been thought to occur in requests for information from a conversational partner [7,11,23–25]. Indeed, eyebrow position is a grammaticalized facial question marker in many sign languages [26–29]. Specifically, in some sign languages [e.g. 30], eyebrow movements can fulfil an important conventionalized signalling function in a particular type of question context—so-called ‘other-initiated repair’—which is core to the process of ‘grounding’ and coordination during dialogue [31,32], i.e. the process of establishing the mutual belief that communicative acts have been understood well enough for current purposes [33,34]. Other-initiated repair (OIR) is a brief exchange between interlocutors that momentarily interrupts the progress of a conversation to solve a communicative problem [35–37] (a phenomenon that has been termed ‘clarification requests’ elsewhere [38,39]). An OIR sequence consists of a *repair initiation*, i.e. a signal from the addressee indicating a problem in perceiving or understanding what the speaker just said, and a *repair solution*, which involves the speaker repeating part or all of the trouble source turn, providing an elaboration or reformulation to clarify certain parts of it, or confirming or disconfirming a candidate understanding offered by the addressee [35,37,39–41].

Judging from their linguistic functions in signed languages, eyebrow raises and furrows may also be normative practices in *spoken* OIR. While repair can be initiated and resolved verbally in absence of the visual channel (think of speaking on the telephone), in spoken face-to-face conversation, eyebrow movements have also been observed in OIR contexts [42] (as well as other visual signals such as manual gestures [43–47], torso movements [48–50], head movements and facial signals [51–54], or the temporary abandoning of all movement [54,55]; see [56] for a summary of visual signalling and repair). An open question is whether eyebrow movements play a communicative role in initiating repair in spoken language. Specifically, an important question is whether they might be epiphenomenal, that is, mere correlates or ‘ornaments’ of verbal initiations without a signalling function in their own right, or whether they are perhaps not communicatively intended but at least perceived in such a way.

In the present article, we address this issue, presenting two studies investigating the use and communicative consequences of eyebrow movements as potential signals of communicative problems in face-to-face interaction. In study 1, we remain on the descriptive side (without being able to determine why behaviour is produced or how it is perceived), and in study 2, we test the perception of eyebrow movements occurring with conversational repair (but not the question as to whether, or how, they are intended by the brow action producer). More specifically, in study 1, we collected data from face-to-face Dutch spoken conversations, coded the co-occurrence of eyebrow movements (raises and furrows) with different types of verbal signals of problems in hearing or understanding (i.e. OIRs), the temporal relationship between the visual and verbal components in these instances, as well as the type of repair solutions provided in response. We also make some preliminary observations about cases in which eyebrow movements alone appear to have been treated as signals of problems in hearing or understanding. Based on the correlational evidence from study 1, study 2 experimentally tests the possibility that addressee brow furrowing indeed serves a communicative function in conversation, silently signalling ‘I have not received sufficient information for current purposes’. It does so by manipulating the occurrence of eyebrow movements in a virtual character and measuring the communicative consequences that result from the perception of addressees’ brow furrows in terms of speakers’ verbal behaviour. Taken together, the two studies present converging evidence from a conversational corpus study and an experimental study suggesting that eyebrow movements indeed play a functional role in signalling communicative problems in interaction and that speakers are sensitive to addressee brow furrows, apparently interpreting them as requests for clarification or elaboration. The manuscript thus combines two traditionally quite different approaches (corpus analyses and experimentation), and it advances on extant literature by studying repair—a mechanism core to human communication—from a multimodal rather than just verbal perspective. Moreover, while many studies of language or emotion communication traditionally focus on either the sender or the receiver, the present approach includes both to shed light on the form and function of signals in social interaction. As such, it is in line with recent notions of facial signals as tools for socially contingent actions rather than static expressions [14] and studying specific signals and their detailed multimodal composition, themselves, as well as recipients’ responses to these signals [57].

## 2. Study 1

### 2.1. Eyebrow movements as signals of communicative problems in face-to-face conversation: a corpus study

A few studies provide initial clues that eyebrow movements may not be epiphenomenal in spoken OIR [52,55,58]. Comparing OIR sequences in unrelated spoken and sign languages (northern Italian, Cha’palaa, Argentine sign language), Floyd *et al*. [55] showed that if a repair initiation was accompanied by a bodily ‘hold’, that is, if body movements like eyebrow movements (but also, e.g. hand gestures or head movements) were ‘temporarily and meaningfully held static’ (ibid., p. 1), this hold was often associated with communication problems and not disengaged from until the communication problem was solved. Floyd *et al*. interpreted these holds as displaying that a repair solution is still expected, whereas disengaging from a hold displays that this is no longer so because the problem has already been repaired. Note that Floyd *et al*. [55] did not distinguish between different types of brow movements such as furrows versus raises, though. Furthermore, two individual descriptive examples—one from English (‘raises her eyebrows, pulls down the corner of the mouth’ [52, p. 11]) and one from Siwu (‘puzzled look: furrowing of eyebrows’ [58, p. 238])—suggest that facial signals including eyebrow raises or furrows may be treated as repair initiations without relying on accompanying verbal material. While these studies suggest that eyebrow movements may serve a communicative role in initiating repair, more systematic evidence for spoken language is needed. Furthermore, little is known about the different compositions of repair initiations used in spoken language (e.g. verbal signal with versus without eyebrow movement, or timing of the visual and verbal components) and about the functions of different types of eyebrow movements, such as brow raises and furrows.

Darwin [7] proposed in his principle of antithesis that two opposed movements are likely to develop distinct communicative functions. Eyebrow raises and furrows are formally opposed, constituting two maximally contrastive extremes of how eyebrows can move. They have distinct effects on vision (seeing more versus seeing less [7,59]), and, as mentioned above, they have been associated with emotions of opposed valence [8]. Assuming that eyebrow movements have a signalling function, this raises the question of whether raises and furrows may also serve distinct communicative functions in signalling problems of perceiving or understanding in spoken face-to-face conversation. In Dutch sign language (Sign Language of the Netherlands (NGT) [27]), eyebrow raises mark polar questions (e.g. ‘you mean John?’) and eyebrow furrows mark content questions (e.g. ‘who?’). If the eyebrow actions in information requests in spoken Dutch are akin to the grammatically obligatory use of eyebrow actions in information requests in Dutch sign language, one may expect that in spoken Dutch, eyebrow raises may be more often involved in repair initiations that make confirmation or disconfirmation relevant (e.g. ‘you mean John?’) and eyebrow furrows more often in those that make clarification relevant (often including content question words, e.g. ‘who?’). While they may then not be marking contrastive extremes like positive versus negative emotions, the different types of brow action may then make different types of repair solutions relevant. Moreover, while the type of brow movement involved in a multimodal repair initiation may affect which type of repair solution is provided in response, the mere presence of the brow movement and the timing of the brow movement relative to the verbal repair initiation may affect the speed with which a repair solution is provided. If the addressee’s brow movement is initiated before the verbal signal, it may ‘forewarn’ the speaker about a communicative problem, providing them with a timing advantage when planning an appropriate response (see also [60] on how turn-opening frowns can anticipate utterances involving some kind of trouble, e.g. epistemic challenges). They may thus fulfil a predictive function with potential benefits regarding the fast-paced temporal organization of turns in face-to-face interaction [61] and the presumed desire to address problems of communication quickly.

In study 1, we hypothesize that eyebrow actions contribute to signalling problems of perceiving or understanding not only in sign language but also in spoken language, on the grounds that spoken languages also strongly rely on the visual channel, at least in face-to-face contexts (e.g. [3,5,61–63]). We also hypothesize that eyebrow raises and furrows may serve different functions in signalling problems of perceiving or understanding and that their timing may matter. Specifically, we predict (although to some extent based on sign languages):

- a systematic association between verbal repair initiation format and type of eyebrow action (e.g. open requests and brow raises, restricted requests and brow furrows);
- the type of eyebrow action used with verbal repair initiations to be systematically associated with the type of repair solution provided in response (e.g. confirmation versus clarification); and
- repair time to be reduced by the presence of an eyebrow action or by an eyebrow action produced as a preliminary to verbal repair initiations.

As an exploratory analysis, we were also interested if we would find repair solutions which did not seem to be triggered by verbal repair initiations but by eyebrow movements alone.

To address these issues, we used data from dyadic Dutch face-to-face conversations, which were specifically designed for detailed analyses of facial behaviour. We identified OIR sequences in conversations and coded the compositionality of repair initiations, focusing on eyebrow raises and furrows. We then quantified the co-occurrence of different linguistic formats of verbal repair initiations with eyebrow raises and furrows, the temporal relationship between the visual and the verbal component in the multimodal repair initiations, and investigated whether the presence of eyebrow actions in general and early eyebrow actions in particular (produced as preliminaries to verbal repair initiations) sped up the repair process. Finally, we made some preliminary observations regarding silently produced addressee eyebrow raises and furrows that seemed to be treated as making repair relevant despite the absence of on-record verbal repair initiations.

## 3. Methods: study 1

### 3.1. Participants and corpora

We used three corpora of spontaneous, dyadic Dutch face-to-face conversations: the Institute of Phonetic Sciences Amsterdam University Dialog Video corpus (IFADV [64]), the purpose-built corpus of Dutch Face-to-Face (DF2F) conversation (see also [65]) and the CoAct corpus (forming part of European Reseach Council project no. 773079 led by J.H.; see also [66–68]). All three corpora were specifically designed to allow for detailed analyses of communicative facial behaviour.

The IFADV corpus consists of 23 dyads, all native Dutch speakers (12–72 years) who knew each other well prior to the recording. Nine of the dyads consisted of a female and a male participant, 11 were all female and three were all male. Five of the participants participated in two dyads each. The dyads were engaged in spontaneous DF2F conversations for 15 min. Conversations were recorded in a soundproof room, and participants were seated at a table, facing each other, positioned approximately 1 m from each other. Two video cameras (JVC TK-C1480B, 720 × 576, 25 fps) were used to record frontal views of each participant, and audio was recorded using head-mounted microphones (Samson QV).

The DF2F corpus consists of 10 dyads, all native Dutch speakers (18–68 years) who knew each other well prior to the recording. Four of the dyads consisted of a female and a male participant, four were all female, and two were all male. Each participant formed part of only one dyad. The dyads were each engaged in casual face-to-face conversations for 1 h. The recordings took place at the Max Planck Institute for Psycholinguistics in Nijmegen, The Netherlands, in a soundproof room. Participants were positioned approximately 1 m from each other at a 45-degree angle. Three High Definition (HD) video cameras (JVC GY-HM100) were used to record frontal views of each participant and a scene view. Audio was recorded using lightweight head-mounted microphones (DPA-d:fine-88) and an audio recorder (Roland R-44) recorded the two audio tracks in synchrony. Each recording session resulted in three videos and two audio files, which were then synchronized and exported in Adobe Premier Pro CS6 (MP4, 24 fps). Originally, each recording session consisted of three 20 min phases: during one 20 min phase, participants did not wear the head-mounted microphones, and audio was only recorded using a ceiling microphone. During a second 20 min phase, audio was recorded using the head-mounted microphones, and during a third 20 min phase, audio was recorded using the head-mounted microphones, and, in addition, participants wore eye-tracking glasses. To achieve the highest audio quality and to allow for detailed analyses of facial behaviour (without potential occlusion of facial behaviour related to wearing eye-tracking glasses), only the 20 min phase in which participants wore head-mounted microphones was used for this study. Each participant was paid 16 euros for the whole session which lasted about 90 min. The study was approved by the Social Sciences Faculty Ethics Committee, Radboud University Nijmegen, and informed consent was obtained before and after filming.

The CoAct corpus consists of 34 dyads for whom all data streams could be successfully synchronized. All participants were native Dutch speakers (mean age: 23 ± 8 years, 51 females and 17 males) who knew each other well prior to the recording. Each participant formed part of only one dyad. The dyads were each engaged in casual face-to-face conversations for 1 h (20 min entirely unprompted, 20 min free conversation about a set of discussion topics (data privacy, use of social media and language of university education) and 20 min in a collaborative planning task). The recordings took place at the Max Planck Institute for Psycholinguistics in Nijmegen, The Netherlands, in a soundproof room. Participants were positioned facing each other, at approximately 90 cm distance. Two video cameras recorded frontal head shots of each participant at 25 fps (Canon XE405). Further video cameras recorded whole-body views and the scene as a whole, but for the purpose of the current study, only the close-up face recordings were used since they provided the best view of detailed facial signals. Audio was recorded using two directional microphones (Sennheiser me-64). The video files and audio files were synchronized in Adobe Premiere Pro CS6 (exported as a single audio-video MPEG file per session, 25 fps). The corpus study was approved by the Social Sciences Faculty Ethics Committee, Radboud University, Nijmegen.

### 3.2. Analysis

We identified occurrences of OIR and eyebrow raises and furrows, sampling from randomly selected 10 min segments in the IFADV corpus (one segment per dyad, resulting in 230 min) and from naturally occurring tellings [69] in the corpus (all tellings in all dyads, resulting in 68 min), resulting in a total of 298 min of conversation. The choice to sample from randomly selected segments in the IFADV corpus and tellings was based on practical considerations. OIR cases were already partially coded in the IFADV corpus (by P.H.), and brow movements were already partially coded in tellings of the DF2F corpus (by P.H.). These annotations were created in ELAN 4.8.1 [70].

In a second bout of gathering OIR cases to lead to more rigorous statistical analyses, we annotated data in the third corpus described above. In connection with a larger project, the corpus had been annotated for all question–response sequences and the social actions these performed [66,67]. Part of these social actions were OIRs, which were also used in the present analysis. Annotations were created in ELAN 5.5.

### 3.3. Other-initiated repair

We first focused the analysis on verbal cases of OIR, i.e. interactional sequences in which one participant signalled trouble in understanding, thus initiating repair on a trouble source located in the prior turn and resulting in a repair solution from the participant producing that prior turn [35,36,40,41]. To be clear, the term ‘repair initiation’ thus refers to the current *addressee* requesting repair. The current *speaker*’s attempt to provide the repair is referred to as the ‘repair solution’. For each OIR case, the linguistic format of the verbal repair initiation, as well as non-mutually exclusive characteristics of the verbal repair solution, were coded.

Three basic formats of **repair initiations** were distinguished [40,41,71]: a repair initiation was coded as (i) open class repair initiation, or *open request,* if it targeted the prior turn as a whole (e.g. *huh?, what?*), typically making repetition relevant but sometimes also clarification, (ii) as a *restricted request* if it targeted a specific aspect of the prior turn (e.g. *who?*), making a clarification of this aspect relevant, and (iii) as *restricted offer* if it targeted a specific aspect of the prior turn by offering a candidate understanding (e.g. *you mean John?*), making confirmation or disconfirmation relevant.

For each **repair solution**, it was coded whether any material from the trouble source turn was (i) repeated, (ii) clarified, or whether (iii) it included a confirmation or disconfirmation (non-mutually exclusive options). A repair solution was coded as ‘repeating’ if some or all material from the trouble source turn was repeated [72], not taking into account whether ‘dispensable’ items such as a turn-initial *but* or *oh* [73] were omitted or not. A repair solution was coded as ‘clarifying’ if it involved modification or specification of the trouble source [74], that is, if some or all material from the trouble source was rephrased, replaced or if something was added. A repair solution was only coded as ‘(dis)confirming’ if it included a “yes/no/indeed” type item, a head nod/shake or a repetition (± negation)’ [75, p. 42], often produced in response to an offered candidate understanding as part of the repair initiation [35,73]. Note that a repair solution was not coded as ‘(dis)confirming’ if it included an indirect (dis)confirmation, for example, by offering an alternative.

Criteria for identifying and classifying OIR cases were based on a coding scheme developed by [75]. In the IFADV corpus and the DF2F corpus, all repair sequences were identified by the first author (P.H.) experienced in the reliable application of this coding scheme through participation in earlier projects on verbal repair. These annotations from the IFADV and DF2F corpora resulted in a total of 118 OIR cases. In the CoAct corpus, a total of 486 OIR sequences were identified. The repair sequences were identified by a group of coders who were trained in coding a wider range of social actions (for an unrelated project), including OIR, applying the same coding scheme as for the other two corpora. Reliability for the coding by P.H. was trained in association with other projects on verbal repair. Reliability for the coders of the CoAct corpus was established involving 32.9% of the question data. This resulted in a raw agreement of 76% (for more details, see [66,67]). The specific linguistic repair initiation and repair solution formats were coded by another two coders (A.G. and L.v.O.), blind to any hypotheses, and resulted in high agreement (based on *n* = 95 OIR cases), namely 92% raw agreement and a Cohen’s kappa of 0.87 for repair initiation formats, and 91% raw agreement and a Cohen’s kappa of 0.87 for the repair solution formats, indicative of substantial agreement [76,77].

In total, 592 OIR cases were identified across the three corpora, originating from 108 speakers in 62 dyads. Of these 592, 18 cases were excluded from further analyses owing to the cases qualifying as two rare linguistic formats of restricted OIR: alternative questions (which invite a selection from several alternatives) and external repair initiations (which address problems about unexpressed elements in the trouble source); one case was ambiguous in terms of OIR modality categorization. The 18 exclusions resulted in a total of 586 OIR cases that were included in the analyses.

#### 3.3.1. Eyebrow actions

We identified eyebrow raises and furrows (facial AU1 + 2 and 4, respectively [21]), annotated from the first to the last frame of visible movement of the eyebrows. This was always a secondary step, i.e. following the identification of OIR, meaning our dataset consisted only of eyebrow actions that accompanied (or initiated) OIR sequences. In the IFADV corpus and the DF2F corpus, eyebrow actions were identified by two independent coders (K.K. and M.K.) who were blind to the hypotheses. Twelve minutes were coded for training and 59 randomly selected minutes (approx. 20% of the total data) were coded for measuring inter-rater reliability. The inter-rater reliability was 76.5% for brow action occurrence, and a Cohen’s kappa of 0.88 was achieved for agreement about the brow action type (brow furrow versus brow raise) indicating substantial agreement. In the CoAct corpus, eyebrow actions were identified by a team of coders annotating all visual communicative facial signals in connection with another project, leading to good agreement (76%; see [67] for more detail).

#### 3.3.2. Compositionality of repair initiations

For each repair sequence, we assessed whether the verbal repair initiation co-occurred with eyebrow actions or not. If they did, we assessed the temporal relationship between the visual and the verbal components. Eyebrow actions were considered to be ‘co-occurring’ if they temporally overlapped with a verbal repair initiation, or if the offset or onset of the eyebrow action immediately preceded or followed the onset or offset of the verbal repair initiation without perceptible interruption, such that the behaviours together formed a multimodal *Gestalt* [61,78]. More precisely, if the onset of the verbal repair initiation and the onset of the eyebrow action coincided precisely or if the onset of one preceded the onset of the other by less than 200 ms, this was coded as ‘initiated simultaneously’. If the onset of the eyebrow action preceded the onset of the verbal repair initiation by more than 200 ms, this was coded as ‘initiated visually first’ (or ‘verbal OIR with visual preliminary’, see §4.4), and if the onset of the verbal repair initiation preceded the onset of the eyebrow action by more than 200 ms, it was coded as ‘initiated verbally first’. While the 200 ms offset is to some extent arbitrary, it corresponds to the time window in which visible lip movements and articulatory sounds [79] are perceived as synchronous; it is close to the natural offset that characterizes the timing of words and meaningful gestural movements in conversational speech [80] and their integration during comprehension [81].

#### 3.3.3. Eyebrow actions occasioning repair in the absence of vocalization

Finally, we also identified repair solutions that appeared to be occasioned by eyebrow actions alone, that is, without any ‘on-record’ verbal repair initiation (e.g. [52]), and were thus coded as ‘eyebrow actions only occasioning repair’. However, please note that, owing to feasibility, we did not annotate all eyebrow actions in the three corpora, but only those cases that occurred in connection with repairs. For this reason, the analysis must be considered preliminary, as we do not have data that would provide insight into how often eyebrow actions were intended to initiate repair when they occurred without verbal repair initiations but did not lead to repair solutions.

### 3.4. Statistical analysis

All analyses were computed with *lme4* (v.1.1-34) [82] in R (v.4.3.1, R Core Team) [83], and all model comparisons with the *anova* function [84].

First, to statistically test whether the *presence of eyebrow action* in repair initiations (verbal-only repair initiation and verbal repair initiation with eyebrow action) was associated with the linguistic *format of the verbal repair initiation* (open request, restricted request and restricted offer), a mixed-effects logistic regression analysis was performed (including random intercepts for participants). An intercept-only model with ‘presence of eyebrow action’ as a dependent variable was compared to a model in which ‘linguistic format of verbal repair initiation’ was added as a predictor variable, using a likelihood ratio test.

Second, to test whether the *type of eyebrow action* in repair initiations (verbal repair initiation with eyebrow raise and verbal repair initiation with eyebrow furrow) was associated with the linguistic *format of the verbal repair initiation* (open request, restricted request and restricted offer), an additional mixed-effects logistic regression analysis was performed (including random intercepts for participants). Again, an intercept-only model with ‘type of eyebrow action’ as a dependent variable was compared to a model in which ‘linguistic format of verbal repair initiation’ was added as a predictor variable, using a likelihood ratio test.

Third, to test whether the *presence or type of eyebrow action* in repair initiation predicted the *type of solution* provided, we correlated the composition of the repair initiation (verbal-only repair initiation, verbal repair initiation with eyebrow raise and verbal repair initiation with eyebrow furrow) with different non-mutually exclusive characteristics of the subsequent repair solution, namely whether any material from the trouble source turn was repeated, clarified (i.e. additional information provided) or whether it included a confirmation or disconfirmation. Note that this analysis could not be applied to six OIR cases in which the repair solution was absent.

Fourth, we used a mixed-effects logistic regression analysis (including random intercepts for participants) to test whether the *composition of the repair initiation* (verbal-only repair initiation, verbal repair initiation with eyebrow raise and verbal repair initiation with eyebrow furrow) predicted whether the *repair solution* included a clarification or not (clarification and no clarification) while taking into account variability in the linguistic format of the verbal repair initiation (open request, restricted request and restricted offer) by adding it as a predictor variable to the statistical model. This model was compared to a reduced model without the predictor variable of ‘composition of repair initiation’ using a likelihood ratio test.

Fifth, we tested in a mixed-effects model whether *repair time* differed between verbal repair initiations *with versus without a brow action* while taking into account variability in the linguistic format of the verbal repair initiation by adding it as a predictor variable to the statistical model. We entered ‘linguistic format’ (open request, restricted request and restricted offer) and ‘presence of brow action’ (yes, no) as fixed effects and intercepts for participants as a random effect into the model. This model was compared to a reduced model without ‘presence of brow action’ as a fixed effect using a likelihood ratio test.

Finally, we tested in a mixed-effects model whether *repair time* differed between verbal-only repair initiations versus repair initiations with a concurrent eyebrow versus an early eyebrow action, while taking into account variability in the linguistic format of the verbal repair initiation by adding it as a predictor variable to the statistical model. We entered ‘linguistic format’ (open request, restricted request and restricted offer) and ‘brow action’ (verbal-only repair initiation, repair initiation with early brow action and repair initiation with concurrent brow action) as fixed effects and intercepts for participants as a random effect into the model. This model was compared to a reduced model without ‘brow action’ as a fixed effect using a likelihood ratio test.

To test whether differences between the included corpora could account for some of this variability, all final models were subsequently compared to one with ‘corpus’ added as an additional fixed effect.

## 4. Results: study 1

We focus the analysis first on the compositionality of repair initiations, assessing the co-occurrence of different eyebrow actions with different linguistic formats of the verbal repair initiation. We then explore the corpus-based plausibility of whether eyebrow actions might merely be epiphenomena of verbal repair initiations or whether they may contribute to signalling problems in hearing or understanding by examining (i) whether the type of eyebrow action accompanying repair initiation predicts certain types of repair solutions, even after taking into account variability in the co-occurring verbal repair initiation format, and (ii) whether the presence of an eyebrow action as a preliminary to repair initiation speeds up the repair process. Finally, we offer some preliminary analyses regarding eyebrow actions that appear to be sufficient to occasion repair, even in the absence of verbal repair initiations.

### 4.1. Is presence and type of eyebrow action associated with the linguistic format of verbal repair initiations?

Out of all identified verbal repair initiations (*n* = 586), a substantial number co-occurred with eyebrow actions (45.7% (*n* = 268)). Out of those co-occurring with eyebrow actions, about half co-occurred with eyebrow raises (49.6% (*n* = 133)) and the other half with eyebrow furrows (50.4% (*n* = 135)).

**Figure 2.**
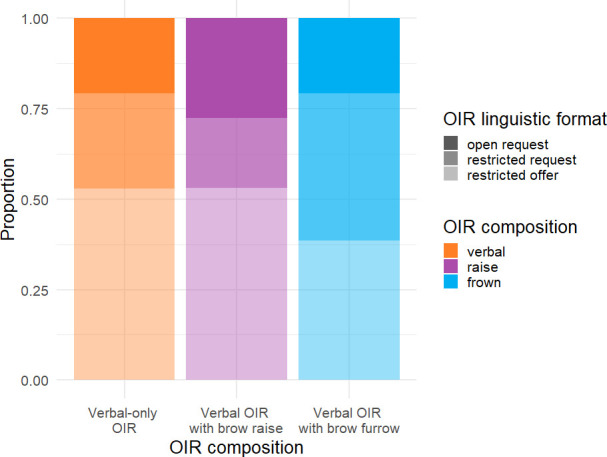
Compositionality of repair initiations (verbal-only OIR, verbal OIR with eyebrow raise and verbal OIR with eyebrow furrow) by linguistic format of the verbal repair initiation (open request, restricted request and restricted offer; *n* = 586).

Which composition (verbal-only repair initiation, verbal repair initiation with eyebrow raise or verbal repair initiation with eyebrow furrow) co-occurred with which linguistic format of the verbal repair initiation (open request, restricted request or restricted offer)? As can be seen in figure 2, restricted offer was the overall most frequent linguistic format of the verbal repair initiation (49.7% (*n* = 291)), followed by restricted request (28.1% (*n* = 165)) and open request (22.2% (*n* = 130)). While the distribution of linguistic formats of the verbal repair initiation is similar when considering just verbal-only repair initiations (restricted offer: 52.0% (*n* = 168); restricted request: 26.0% (*n* = 84); open request: 20.4% (*n* = 66)) and just verbal repair initiations with eyebrow raises (restricted offer: 53.4% (*n* = 71); restricted request: 19.5% (*n* = 26); open request: 27.1% (*n* = 36)), verbal repair initiations with eyebrow furrows show a lower proportion of restricted offers (38.2% (*n* = 52)), but a substantially higher proportion of restricted requests (40.4% (*n* = 55)), relative to verbal-only repair initiations and verbal repair initiations with eyebrow raises. A tabular overview of the frequency data can be found in the electronic supplementary material.

The example below (example 1) illustrates how an eyebrow raise may be used with a restricted offer, which is subsequently confirmed through a head nod:


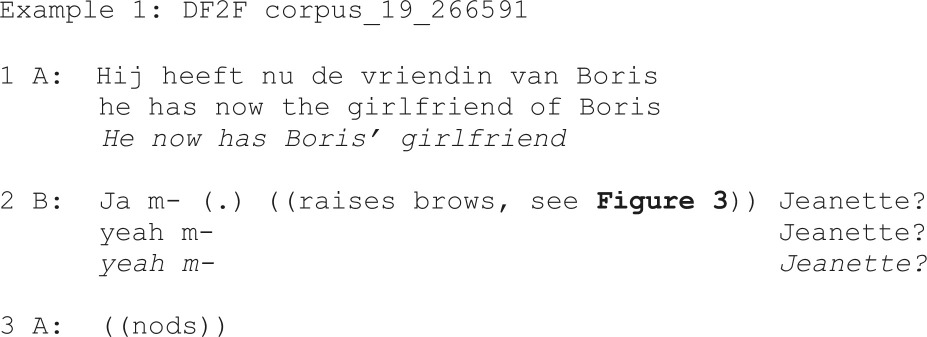


**Figure 3.**
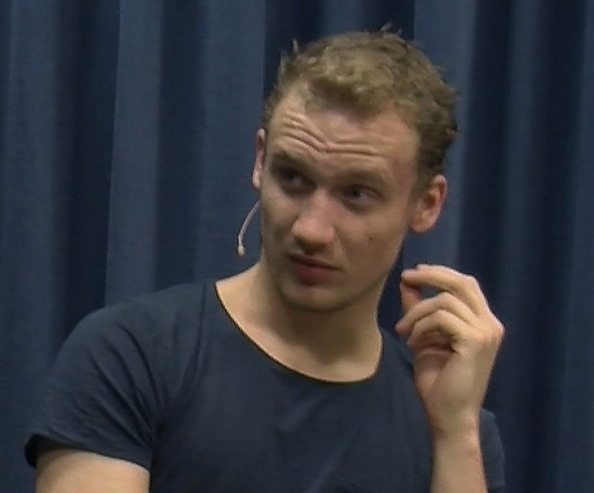
Eyebrow raise produced with a restricted offer as linguistic format of the verbal repair initiation (‘Jeanette?’, line 2 in example 1).

By contrast, example 2 illustrates how an eyebrow furrow may be used with a restricted request, in this case for clarification of an underspecified person reference:


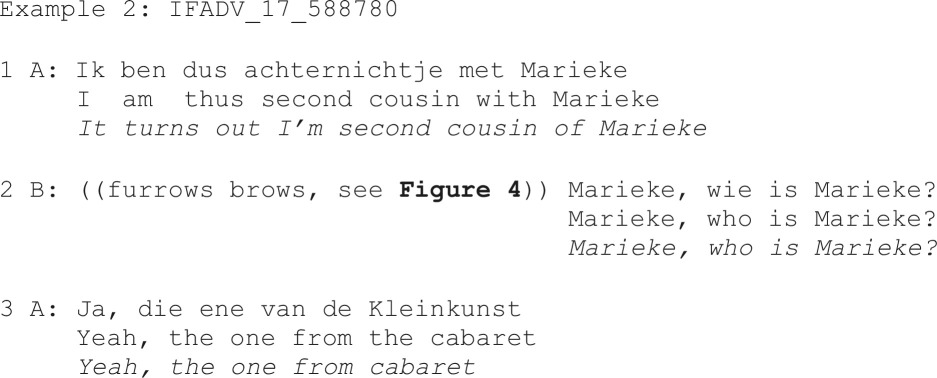


**Figure 4.**
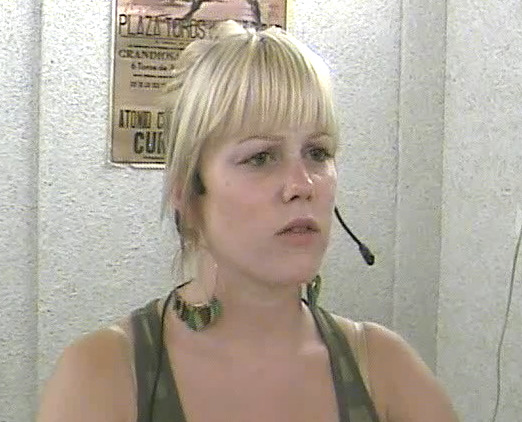
Eyebrow furrow produced with a restricted request as linguistic format of the verbal repair initiation (‘who is Marieke?’, line 2 in example 2).

These observations were tested with model comparisons, which showed that while the *presence of eyebrow action per se* did not improve model fit significantly compared to an intercept-only model (*χ*^2^(2) = 2.74, *p* = 0.254), *type of eyebrow action* did result in significantly improved model fit (*χ*^2^(2) = 14.98, *p* = 0.0006), indicating that type of eyebrow action may distinguish between the linguistic format of the verbal repair initiation.^1^ Post hoc comparisons revealed that the type of brow action is more strongly associated with restricted requests compared to both open requests (log odds ratio = −1.21, standard error (s.e.) = 0.41, *p* = 0.0095) or restricted offers (log odds ratio = −1.23, s.e. = 0.35, *p* = 0.001), with brow furrows occurring more frequently compared to brow raises with this linguistic format. Including *corpus* as a fixed factor did not significantly improve model fit for either *presence of eyebrow action* (*χ*^2^(2) = 1.16, *p* = 0.559), or *type of eyebrow action* (*χ*^2^(2) = 1.56, *p* = 0.458).

### 4.2. Does the presence or type of eyebrow action in repair initiation predict the type of repair solution provided?

To address this question, we related the composition of the repair initiation (verbal-only repair initiation, verbal repair initiation with eyebrow raise and verbal repair initiation with eyebrow furrow) to different non-mutually exclusive characteristics of the subsequent repair solution, namely whether any material from the trouble source turn was repeated, clarified or whether it included a confirmation or disconfirmation. Note that this analysis could not be applied to 95 OIR cases in which a verbal repair solution was absent (leaving *n* = 491).^2^ As a result, the analyses related to repair solutions are based on 107 speakers from 61 dyads.

**Figure 5.**
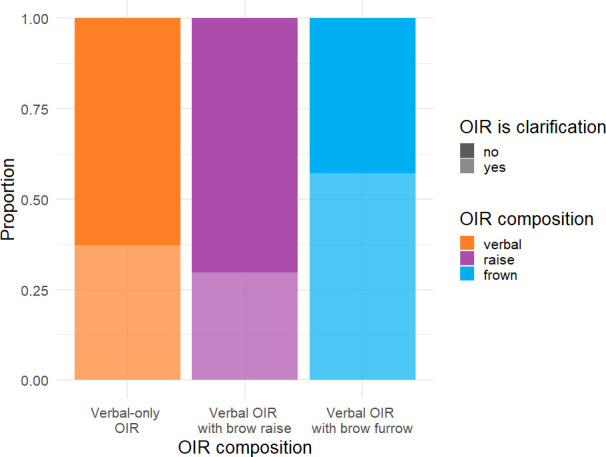
Compositionality of OIR (verbal-only OIR, verbal OIR with eyebrow raise and verbal OIR with eyebrow furrow) by repair solution (yes = with clarification, no = without clarification).

As one can see in figure 5, the percentage of repair solutions that included clarification in response to repair initiations issued with eyebrow furrows was higher (56.3% (*n* = 58)) than the percentage of repair solutions that included clarification in response to verbal-only repair initiations (37.4% (*n* = 104)) and in response to repair initiations issued with eyebrow raises (29.7% (*n* = 33)).

Note, however, that this is not necessarily a unique contribution of eyebrow furrows. One might argue that it is not surprising that repair solutions produced in response to repair initiations with furrows are more likely to include clarification, given that eyebrow furrows are more frequent in restricted requests (see figure 2). The linguistic format of the verbal repair initiation, in this case, ‘restricted request’, rather than the accompanying eyebrow furrow, may thus underlie the increased frequency for repair solutions to include clarifications. To explore this possibility, we used a mixed-effects logistic regression analysis to test whether the composition of the repair initiation predicts whether the repair solution included a clarification or not while taking into account variability in the linguistic format of the verbal repair initiation. A likelihood ratio test showed that *composition of repair initiation* improved the model fit significantly (*χ*^2^(2) = 10.83, *p* = 0.004), revealing an association between the composition of repair initiation and the format of the repair solution. Post hoc comparisons revealed that eyebrow furrows were more strongly associated with clarifications compared to both eyebrow raises (log odds ratio = 1.15, s.e. *=* 0.37, *p* = 0.005) and verbal-only repair initiations (log odds ratio = 0.736, s.e. = 0.30, *p =* 0.04). These results indicate that, independently of the linguistic format of the repair initiation, the presence of an eyebrow furrow increased the likelihood of a repair initiation being treated as a request for clarification. Including *corpus* as an additional fixed effect did not significantly improve model fit (*χ*^2^(2) = 2.46, *p* = 0.293).

### 4.3. Do eyebrow actions speed up the repair process?

**Figure 6.**
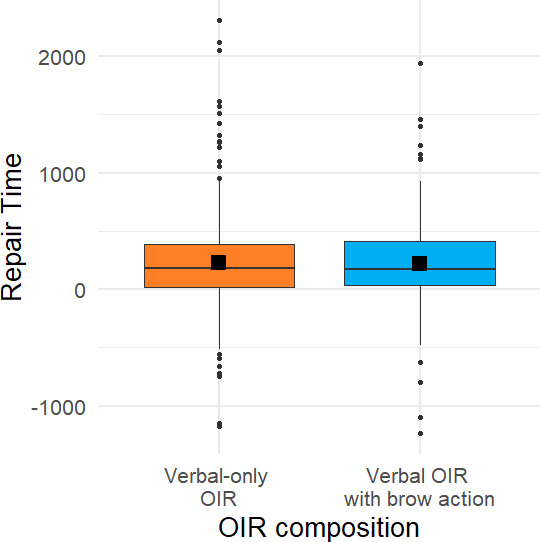
Repair time, measured from the end of the repair initiation until the start of the repair solution, by repair initiation *without* an eyebrow action (verbal-only OIR) versus *with* an eyebrow action (verbal OIR with brow action).

If eyebrow actions were merely a correlate of verbal repair initiation—say a *symptom* of cognitive effort—rather than a communicative *signal* of a problem in hearing or understanding, one should expect the repair time, measured from the end of the repair initiation to the start of the repair solution, to be unaffected by the presence of an eyebrow action. Alternatively, if eyebrow actions can indeed function as a communicative *signal* of a problem in hearing or understanding, one may expect that the presence of an eyebrow action *per se* may reduce potential ambiguity and express a stronger sense of urgency, which may reduce the repair time. To address this issue, we compared the repair time between verbal repair initiations without versus with a brow action (see figure 6). We observed small differences in repair time for different repair compositions, with verbal-only repair initiations having an estimated marginal mean repair time of 241 ± 30.1 ms (s.e.), and repair initiations with brow action 244 ± 32.9 ms (raises: 214 ± 44.2 ms; furrows: 275 ± 45.7 ms). These observations were statistically tested through model comparisons, which showed that neither *presence of brow action* (*χ*^2^(1) = 0.0063, *p* = 0.937), nor *type of brow action* (*χ*^2^(1) = 1.0098, *p* = 0.604) as fixed effects improved model fit when accounting for the linguistic format of repair initiation, thus brow action did not appear to reliably speed up the repair process in the current dataset. Including *corpus* as an additional fixed effect did not significantly improve model fit for either *presence of brow action* (*χ*^2^ (2) = 0.832, *p* = 0.660) or *type of brow action* (*χ*^2^(2) = 0.743, *p* = 0.690).

### 4.4. Does the timing of eyebrow action influence repair time?

**Figure 7.**
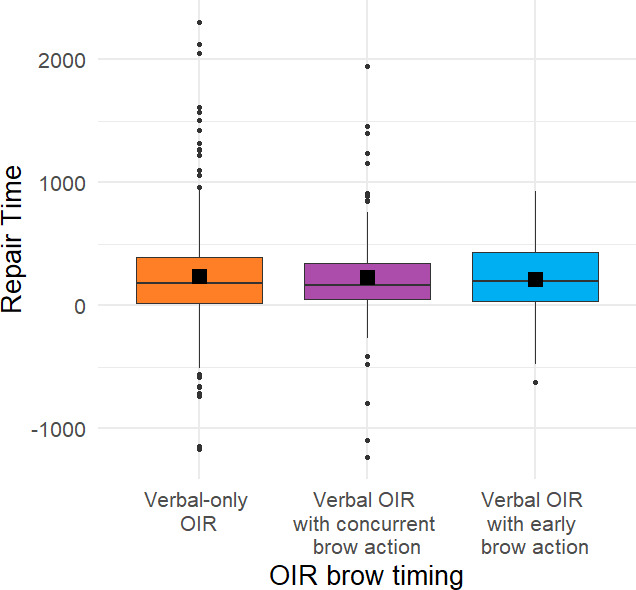
Repair time, measured from the end of the repair initiation until the start of the repair solution, by *verbal-only* repair initiations (verbal-only OIR) versus repair initiations with a *concurrent* eyebrow action (verbal OIR with concurrent brow action) versus an *early* eyebrow action (produced as a visual preliminary to the verbal repair initiation, ‘verbal OIR with early brow action’).

Although the presence of eyebrow actions as such did not reliably influence repair time, early eyebrow actions specifically may serve as a visual preliminary, a visual ‘forewarning’ and may thus facilitate a timely response and, consequently, reduce repair time. To address this issue, we examined the temporal relationship between the visual and the verbal component in multimodal repair initiations (see §3) and then compared the repair time between *verbal-only* repair initiations, verbal repair initiations with a *concurrent* eyebrow action versus verbal repair initiations with an *early* eyebrow action (produced as a visual preliminary to the verbal repair initiation; see figure 7). There were only small differences in the estimated marginal mean repair time depending on the timing of eyebrow action while controlling for linguistic format of the repair initiation (*verbal-only* repair initiations 241 ms ± 30.5 (s.e.), repair initiations with *concurrent* brow action 232 ms ± 44.9, and repair initiations with *early* brow actions 248 ms ± 50.7). Model comparisons revealed no significant improvement of model fit when including the temporal organization as a fixed effect (*χ*^2^(2) = 0.062, *p* = 0.970). Neither *brow action type* (brow raise, brow furrow; *χ*^2^(1) = 1.233, *p* = 0.267) nor *corpus* (*χ*^2^(2) = 0.9, *p* = 0.638) further improved model fit when added as predictors to the statistical model.

### 4.5. Can eyebrow actions alone signal problems in hearing or understanding?

To address this question, we identified all silently produced eyebrow actions that appeared to occasion repair. This resulted in 11 identified eyebrow furrows and zero eyebrow raises. None of these eyebrow furrows were treated as making (dis)confirmation relevant, but *all* of them were treated as making clarification relevant (while three of these were also treated as making partial repetition relevant). Despite these observations resting on a small number of cases, they may be seen as suggesting that eyebrow furrows alone can be sufficient as signalling a need for clarification, even in the absence of verbal repair initiations. Example 3 illustrates how an eyebrow furrow alone seems to occasion repair, similar to a restricted verbal request for clarification.


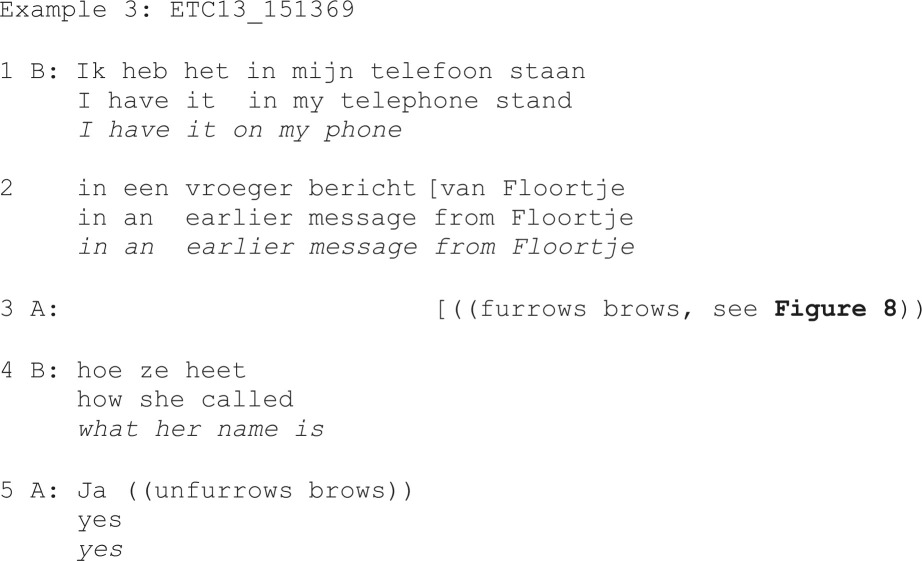


**Figure 8.**
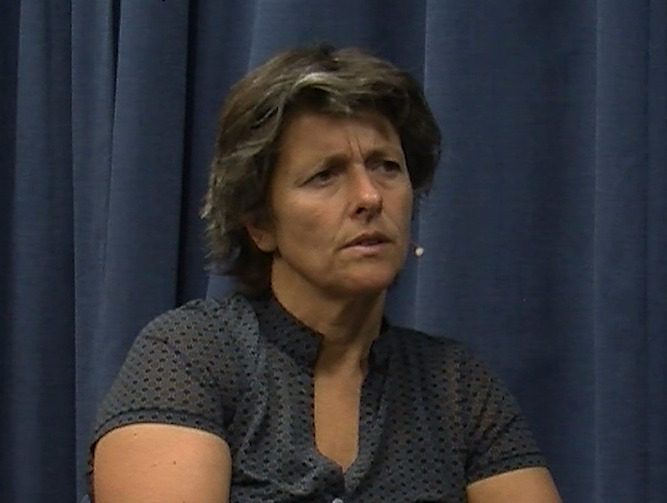
Eyebrow furrow alone occasioning clarification (‘what her name is’, line 4 in example 3).

In the example mentioned above, B targets *it* (‘het’, line 1) as the trouble source by clarifying what *it* referred to through a repair *what her name is* (‘hoe ze heet’, line 4). As such, without any on-record verbal prompting, A’s eyebrow furrow was treated as if A had produced a verbal restricted request like *What do you have on your phone?* (‘Wat heb je in je telefoon staan?’), and this repair solution is acknowledged as satisfactory by A (‘ja’/yes).

In summary, do eyebrow actions contribute to the processes of initiating and receiving repair in spoken face-to-face conversation? While we do not have evidence that the presence of eyebrow movements during verbal repair initiations or the type of eyebrow movement sped up repair time, several findings do demonstrate a potential signalling function of eyebrow actions in the process of OIR. The main findings from study 1 are as follows:

- the type of eyebrow action during repair *initiation* may distinguish between the linguistic format of the verbal repair initiation since brow furrows occurred more frequently than brow raises with restricted requests (compared to both open requests or restricted offers);
- the presence of eyebrow furrows during repair *initiation* is associated with a greater likelihood that the repair *solution* will include a clarification (rather than a simple repeat or a confirmation/disconfirmation) compared to repair initiations without eyebrow actions, or to repair initiations accompanied by eyebrow raises; and
- a small number of initial observations suggests that eyebrow furrows in the absence of verbal repair initiations seem to be able to elicit repair solutions.

## 5. Discussion: study 1

Do eyebrow movements serve a communicative function in signalling problems of hearing or understanding in spoken conversation? The present findings suggest they may do indeed. The results are incompatible with an epiphenomenal interpretation of eyebrow movements, because (i) we found a reliable association between type of brow action and verbal repair initiation format, with brow furrows being more strongly associated with restricted requests; and (ii) in addition to the linguistic format of the verbal repair initiation, the type of co-occurring eyebrow movement independently predicted the type of repair solution. Preliminary qualitative observations also suggest that eyebrow movements alone may be sufficient to occasion clarification.

More specifically, we found that eyebrow raises and furrows were both used with all three basic linguistic formats of repair initiation, whether the co-occurring repair initiation targeted the prior turn as a whole (open request), a specific aspect of it (restricted request), or whether the repair initiation offered a candidate understanding (restricted offer). A higher proportion of eyebrow furrows co-occurred with restricted requests (‘who?’) relative to repair initiations with eyebrow raises, and a higher proportion of eyebrow raises co-occurred with restricted offers (‘John Smith?’) relative to repair initiations with eyebrow furrows—a numerical pattern that parallels the linguistic function of eyebrow position in Dutch sign language, where eyebrow furrows serve as non-manual grammatical markers of content questions and eyebrow raises as non-manual grammatical markers of polar questions [27]. Repair initiations without eyebrow actions and repair initiations with eyebrow raises showed an almost identical distribution regarding restricted offers, potentially pointing to a higher optionality of the use of eyebrow raises in polar questions as repair initiations in spoken face-to-face conversation.

The present study also provides evidence that the type of eyebrow movement co-occurring with repair initiations predicted differences in how these multimodal signals of problems were treated as making relevant different solutions. The presence of an eyebrow furrow uniquely—i.e. independently of the linguistic format of the verbal repair initiation—increased the likelihood of a repair initiation to receive a repair solution including clarification. This suggests that the visual component is not merely a correlate of the verbal component, but that eyebrow furrows can contribute to signalling the type of communicative problem and how it can best be fixed.

Our analyses do not show evidence that the presence of eyebrow actions speeds up the repair time. Numerically, brow raises were associated with slightly shorter repair times compared to brow furrows; however, this difference was not statistically significant. More importantly, there was no difference in repair time between repair initiations with or without brow action, and this was also not dependent on the temporal organization of the repair initiation.

We have also made some preliminary observations suggesting that eyebrow furrows alone can silently signal insufficient understanding. While facial action like the eyebrow furrow is usually not considered to form part of turn-constructional units in the human turn-taking system [85], the present cases suggest that they may serve sequentially equivalent functions to verbal repair initiations. As Levinson [86, p. 74] noted, ‘Words and deeds are the same kind of interactional currency’. However, some might argue that while an eyebrow furrow seems slightly more accountable than a ‘freeze look’ [30], it still may not be considered as explicitly encoding the intention to initiate repair—potentially in an effort to minimize any possible ‘face-threatening’ consequences [87]—‘just as “It’s cold in here” does not explicitly encode the intention to get somebody to shut the window’ [88, p. 11]. To what extent silent eyebrow furrows are treated in interaction as on a par with verbal repair initiations or as a more ‘off-record’ form of communication is an interesting question for future research, but the fact that they seem to be sufficient to elicit repair by themselves points towards the former (which is in line with other non-verbal activities, such as laughter or headshakes [89,90]; see also [91] for reference to brow furrows and repair).

Moreover, purely visible bodily behaviours used to initiate repair have previously been classified as open requests (equivalent to e.g. *huh?*) and thus as not explicitly targeting specific aspects of the trouble source but the trouble source as a whole, typically treated as making repetition relevant (e.g. [37]). However, eyebrow furrows were treated as making *clarification* relevant, specifically targeting certain aspects of the prior turn. If the eyebrow furrow as such signals a need for clarification specifically, it may be easy to guess for the speaker, based on estimates of shared knowledge, which aspect of the prior turn needs clarification (e.g. an underspecified person reference). Alternatively, as with visual addressee signals more generally, since they do not interfere as much with the spoken turn as verbal addressee signals, a specific troublesome aspect of a turn cannot only be targeted through explicit verbal means (e.g. *Who?*) but also through timing. That is, producing the visual signal immediately after the troublesome part (e.g. ambiguous person reference) of the ongoing turn may already signal what part of the trouble source turn needs clarification. In-depth future examinations of the precise temporal relationship between brow movement and trouble source may shed light on this issue.

Note that in the present study, we did not find any eyebrow *raises* that occasioned clarification without relying on a verbal signal, which may in part be explained by the close association of eyebrow raises and speech prosody [92,93]. This does not mean that eyebrow raises can never occasion repair without relying on a vocal signal in spoken Dutch. At least anecdotal evidence suggests that also in spoken Dutch eyebrow raises—especially when combined with a downward movement of the corners of the mouth—can also occasion clarification without a vocal signal, especially after a try-marked person- or place reference. This facial gesture combining brow and mouth actions has been termed a ‘facial shrug’ [13,94]—a signal of ‘not knowing’ [13, p. 15], see also [52, pp. 10−11].

Taken together, the results from study 1 provide correlational evidence regarding the hypothesized involvement of eyebrow movements in signalling problems of hearing or understanding in spoken face-to-face communication. However, because in conversational corpora a multitude of behaviours happen at any given time, controlled experimental work (study 2) is required to provide conclusive evidence about potential causal relations between eyebrow movements and conversational repair.

## 6. Study 2

### 6.1. The cooperative eyebrow furrow: a facial signal of insufficient understanding

Taken together, the results from study 1 suggest a communicative function of addressee’s eyebrow furrows in signalling ‘I’ve *not* received enough information for current purposes’ [33,34]. Based on these correlational findings we ask: is there a causal influence of addressee eyebrow furrows on speakers’ communicative behaviour in face-to-face interaction?

To address this question, we developed an experimental paradigm using virtual reality technology enabling us to selectively manipulate visual feedback in virtual addressees (see also [95–99]). This selective manipulation allowed us to address questions regarding the causal role of eyebrow furrows in interactive face-to-face communication—questions that have previously been impossible to address with such a high degree of experimental control. Participants were asked to have a conversation with different avatars and to answer open questions (e.g. *How was your weekend, what did you do?*). During the participants’ answers, the avatar produced different types of visual feedback responses, which were secretly triggered by a confederate (i.e. a Wizard of Oz paradigm). In one condition, the confederate responses were translated by a script to always trigger nods in the avatar (baseline ‘nod’ condition). In a second condition, the confederate responses triggered nods but, crucially, occasionally an eyebrow furrow instead (experimental ‘nod/brow furrow’ condition). A control condition was identical to the experimental ‘nod/brow furrow’ condition, except that the eyebrow furrows were replaced with no response at all while the nods were retained (control ‘nod/non-response’ condition). This condition was included to control for the fact that there would be fewer nods in the ‘nod/brow furrow’ than in the ‘nod’ condition. Thus, we would be able to tease apart whether any differences between these latter two conditions were owing to the reduction of nods or the presence of eyebrow furrows.

If addressees’ eyebrow furrowing is irrelevant for speakers’ speaking behaviour, one would not expect any differences between the nod condition and the nod/brow furrow condition. However, if addressees’ eyebrow furrowing can indeed signal ‘I’ve not received enough information for current purposes’, providing evidence for unsuccessful grounding [11], speakers should provide clarifying information; that is, they should provide longer answers in the nod/brow furrow condition than in the nod baseline condition. This is the main hypothesis study 2 is testing, based on the logic that if eyebrow furrows are perceived by speakers as requests for more information, then longer answers should indicate that speakers provide additional semantic information to respond to this request. However, there is the possibility that furrowed brows throw speakers off course a little, thus leading to more hesitations than in the other conditions—unfilled, silent pauses and filled pauses like *uh* and *uhm*—which may alternatively explain any differences in overall answer length. To be able to rule out this possibility, we also measured the frequency and duration of filled and unfilled pauses within each answer. Finally, if addressees’ eyebrow furrowing signals a need for further information, then their presence should lead to longer answers also in comparison to the control condition, where eyebrow furrows were replaced with no feedback at all (i.e. no nod, no eyebrow furrow).

Speaking behaviour, like any other social behaviour, varies from individual to individual [100]. In this experiment, two particular individual difference measures of dispositional social sensitivity—the empathy quotient [101] and the fear of negative evaluation scale (henceforth ‘FoNE’; [102])—were hypothesized to modulate the perception of eyebrow movements. Sensitivity to addressees’ eyebrow furrows may depend on the speaker’s degree of empathy, which is the ‘drive or ability to attribute mental states to another person/animal, and entails an appropriate affective response in the observer to the other person’s mental state’ [101, p. 168]. It has been observed that ‘to drive your point home in a discussion for far longer than is sensitive to your addressee’ constitutes low-empathy behaviour [101, p. 170], suggesting that low-empathy speakers may be less sensitive to addressee feedback than high-empathy speakers. To address this issue, participants were asked to complete the empathy quotient questionnaire after the experiment. Sensitivity to addressees’ eyebrow furrows may also depend on the speaker’s degree of FoNE. In contrast with low-FoNE individuals, high-FoNE individuals are highly concerned with seeking social approval [102]. High-FoNE individuals have been shown to exhibit more pro-social behaviour [103] and try harder to make a good impression during face-to-face conversations [104]. According to Leary [104, p. 371], ‘People who are highly concerned about being perceived and evaluated negatively would be more likely to behave in ways that avoid the possibility of unfavourable evaluations and, thus, be more responsive to situational factors relevant to such concerns than individuals who are less apprehensive about others’ evaluations of them’. One such relevant situational factor may be others’ facial expressions. Indeed, high-FoNE individuals have been shown to pay more attention to faces [105], particularly to faces expressing negative emotions owing to their potentially socially devaluating meaning [106,107]. Since eyebrow furrowing is associated with expressions of negative emotions like anger [8], one might expect high-FoNE individuals to be especially sensitive to addressees‘ eyebrow furrows as they occur in the present study. Finally, high-FoNE individuals have also been shown to judge their own communicative effectiveness more accurately, that is, in a way that is more consistent with addressee’s actual understanding, which might be owing to their increased sensitivity to addressee feedback [108].

If addresses’ eyebrow furrows are not a semiotic, conventional signal but, for example, a symptom of cognitive effort, one may expect only high-empathy or high-FoNE speakers to be responsive to addressees’ eyebrow furrows in the messages they design, owing to their stronger social sensitivity. However, if addressees’ eyebrow furrows are indeed a semiotic, conventional signal, one may expect all speakers to be sensitive to addressees’ eyebrow furrows (although high-empathy or high-FoNE speakers even more so).

The overall aim of study 2 was to experimentally test the claims based on correlational evidence, suggesting that addressee eyebrow furrows may serve a communicative function in conversation (study 1). The main hypothesis was that addressees’ eyebrow furrows can function as a communicative signal of insufficient understanding, that speakers would produce longer answers in the nod/brow furrow condition than in the nod baseline or control condition, while individual differences in speakers’ social sensitivity may modulate this effect.

## 7. Methods: study 2

### 7.1. Participants

We recruited 36 native Dutch speakers through the Max Planck Institute for Psycholinguistics subject database for participation in the experiment. The data of one participant were excluded from all analyses because he provided such long answers to the avatars’ questions that we had to interrupt him and end the experiment prematurely in order to be able to test the remainder of the scheduled participants. The data of one additional participant were excluded from all analyses because he excessively looked away from the screen (more often than 2.5 s.d. above the mean). Another participant did not complete the empathy quotient questionnaire and was therefore excluded from any analyses including the empathy quotient. This resulted in a final sample of 34 participants (18–33 years; mean age = 22.47; 18 females and 16 males) or 33 participants for analyses including the empathy quotient (18–33 years; mean age = 22.54; 18 females and 15 males). Each participant was paid 10 euros, and the whole session lasted about 1 h.

### 7.2. Design

We used a within-subject design with avatar addressee feedback (nod, eyebrow furrow and non-response) as the independent variable and mean answer length as the main dependent variable. Additional variables consisted of the empathy quotient [101], the FoNE score [104], hesitations (frequency and duration of filled and unfilled pauses), as well as avatar evaluation questionnaire scores assessing perceived humanness, ease of understanding by the avatar of the participant and likability of each avatar (see below for details on the questionnaires).

The experiment consisted of three blocks, one block per addressee feedback condition (i.e. one per avatar, see below). The set of 18 spoken question stimuli was split up into three sets of six questions, and each set was assigned to one of three avatars, meaning each participant heard each question only once. The order of addressee feedback conditions as well as the assignment of avatars (and thus the six questions that were paired with the respective avatars) to the addressee feedback conditions was counterbalanced across participants. The order of items within each block was randomized.

### 7.3. Apparatus and materials

#### 7.3.1. Laboratory set-up and equipment

Participants were invited to the Virtual Reality laboratory at the Max Planck Institute for Psycholinguistics in Nijmegen, The Netherlands. They were seated in front of a computer screen (HP Compaq LA2405WG) with speakers (Hercules XPS 2.010) wearing a lightweight, head-mounted microphone (DPA-d:fine-88). Audio was recorded using Adobe Audition CS6, and video was recorded using three synchronized video cameras (Sony 3CCD Megapixel) to capture the participant frontally and laterally, as well as to record a separate computer screen showing exactly what the participant was seeing on their screen (i.e. the avatar). This set-up allowed us to link participant and avatar behaviour in a time-aligned manner. For each recording session, we synchronized the three videos and the audio file based on audible and visible markers (produced at the beginning of each block) and exported them in Adobe Premier Pro CS6 (MP4, 25 fps). The confederate was seated in the control room next to the experiment room, in front of a keyboard (Apple MB110LL/B) and a computer screen (Acer AL732). The computer screen showed the participant in real time from a frontal view. Audio from the participant’s microphone was also transmitted to the control room and played via speakers (Alesis M1Active 520) in real time. The confederate was thus responding to an interaction partner (i.e. the participant) who they saw and heard, but instead of producing visual and vocal addressee behaviour, the confederate was asked to press a button whenever they felt feedback should naturally occur. These button-press responses were translated into different forms of avatar feedback behaviour (see below). Importantly, based on this manipulation, the avatars’ feedback appeared naturally timed. Moreover, to make sure that the confederate behaved consistently, we compared the confederate’s button press behaviour across conditions: predicting the confederate’s feedback button press frequency (number of button presses per answer divided by the length of the same answer in minutes; *M* = 10.74; s.d. = 3.52) by feedback condition (nod, nod/brow furrow and nod/non-response), including random intercepts for participants and items, confirmed that button press frequency was consistent across conditions (nod versus nod/brow furrow: *β* = −0.05*,* s.e. = 0.25, *t =* −0.255*, p* = 0.822; nod versus nod/non-response: *β* = 0.231*,* s.e. = 0.253, *t =* 0.913*, p* = 0.362; nod/brow furrow versus nod/non-response: *β* = 0.288*,* s.e. = 0.253, *t =* 1.138*, p* = 0.256).

#### 7.3.2. Avatar characteristics and behaviours

**Figure 9.**
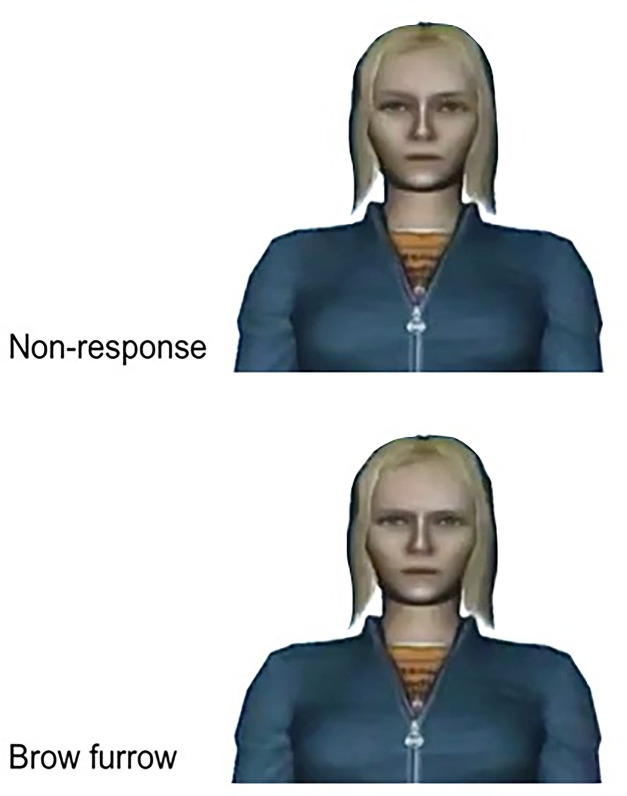
Example stills of a virtual addressee producing different types of addressee feedback responses (non-response and brow furrow) that were varied across conditions.

The experiment was programmed in WorldViz’s Vizard 5.5, and three different female avatars were created based on a stock avatar produced by WorldViz (see figure 9 for an example). Three different female Dutch native speakers were used to pre-record the avatars’ speech, which was played at appropriate times during the experiment (one per condition). The avatars’ lip movements were programmed to match the amplitude of the pre-recorded speech files (i.e. the higher the amplitude, the wider the avatar opened her mouth), creating an illusion of synchronization. The speech materials consisted of a general introduction (e.g. *Hoi, ik ben Julia, leuk je te ontmoeten!*; ‘Hi, I’m Julia, nice to meet you!’ and *Ik heb een aantal vraagen voor jou; ‘*I have a couple of questions for you’) and a set of 18 open-ended questions (e.g. *Hoe was je weekend, wat heb je allemaal gedaan?*; ‘How was your weekend, what did you do?’). The avatar also responded to the participant’s answer (e.g. *Oh ja, wat interessant!*; ‘Oh, how interesting!’) before moving on to the next open question (e.g. *Ik heb nog een vraag voor jou;* ‘I have another question for you’), or before closing the interaction (*Hartelijk bedankt voor dit gesprek, ik vond het gezellig!*; ‘Thank you very much for this conversation, I enjoyed it!’). All of the questions and responses were triggered by the confederate.

All visual feedback responses of all three avatars were triggered secretly by a confederate, a Dutch native speaker who could see and hear the participant (via a video-camera link, see above), who was blind to the experimental hypotheses (and not informed about the manipulations) and who was instructed to imagine being the actual addressee interacting with the participant and to press a button whenever it felt appropriate and natural to provide addressee feedback (or the next question, see above). Which of the confederate’s button presses triggered a nod and which brow furrow (within the nod/brow furrow condition) was varied automatically by the computer program. To avoid unnatural repetitions of eyebrow furrows, we made sure that following each eyebrow furrow, the next one or two feedback responses (randomly varied) would be a nod before the next eyebrow furrow could be produced. In total, the program generated 1497 visual responses. In the nod/furrow condition, the relationship was *n* = 865 (nods) to *n* = 632 (furrows).

The avatar’s gaze behaviour was not varied. Gaze was directed towards the participant at all times, and this was not deemed to be unnatural. While it has been claimed that in human–human interaction listeners do look at speakers much of the time but not continuously [109], more fine-grained analyses of conversations have shown that interactants’ gaze behaviour depends to a large extent on the (sequences of) actions performed [110,111]. Tellings and question–response sequences in particular can at times involve more continuous gaze from the recipient or questioner. Since the avatars asked questions that elicited short tellings from the participants, these forms of action map most closely to the avatar experimental set-up and in natural discourse can receive close to 100% of gaze from the recipient [111]. Especially in the absence of parallel activities and a seating arrangement that favours face-to-face orientation, continuous gaze is unlikely to appear unnerving.

The crucial experimental manipulation in the present study was the feedback responses the avatar produced when she was in the addressee role (see figure 1 for example stills). Critically, the form of these feedback responses was modelled on feedback behaviour that occurs in natural conversation and they consisted of head nods (duration of 500 ms from nod onset to nod offset, all rendered in exactly the same manner, including speed and size of the movements) and eyebrow furrows (duration of 500 ms from eyebrow furrow onset to eyebrow furrow offset, also all rendered in exactly the same manner, including speed and size of the movements). In the control condition, the avatar produced ‘non-responses’, periods in which the avatar did not produce any feedback response. That is, during a ‘non-response’, the avatar was just still (default behaviour). Note that the duration of ‘non-responses’ matched the durations of the other feedback responses precisely (i.e. 500 ms). The timing of these feedback responses was based on the confederate’s behaviour, as described above.

#### 7.3.3. Questionnaires

The questionnaires used in this study consisted of the Dutch version of the Empathy Quotient (EQ) questionnaire (test–retest reliability: *r* = 0.97, as reported by [101]) and the Dutch version of the brief ‘FoNE scale’ (test–retest reliability: *r* = 0.75, as reported by [104]). To control for the possibility that any differences in answer length might be driven by differences in perceived naturalness, perceived ease of understanding by the avatar of the participant and perceived likability of the avatars depending on the different feedback behaviours they produced, we asked participants to fill in three additional questionnaires tapping these three aspects (one for each avatar the participants interacted with). The avatar evaluation questionnaires consisted of statements designed to assess the participants’ perception of the avatars’ (i) humanness (*Ik vond deze avatar menselijk overkomen*; ‘This avatar appeared human’), (ii) ability to understand the participant easily (*Ik denk dat deze avatar mij makkelijk te begrijpen vond*; ‘I think this avatar found me easy to understand’), and (iii) likability (*Ik vond deze avatar sympathiek overkomen*; ‘This avatar appeared nice’; *Ik zou vrienden kunnen zijn met deze avatar*; ‘I could be friends with this avatar’; *Ik vond deze avatar egocentrisch overkomen*; ‘This avatar appeared selfish’) as their conversational partner (adapted from [112]), and the Dutch translations used in the Relationship Questionnaire [113]. Participants indicated on a 6-point Likert scale their degree of agreement for each statement (1 = *I do not agree at all*, 6 = *I absolutely agree*). Statistical tests confirmed that the perceived humanness, ease of understanding by the avatars and likability (rated through scores for niceness, friendship and selfishness, see above) of the avatars did not differ across addressee-feedback conditions (see the electronic supplementary material).

### 7.4. Procedure

Participants were seated in front of the computer screen and were asked to meet and have a conversation with three different avatars by responding to their questions. After a short personal introduction, the avatar asked questions and produced different types of visual feedback responses while participants answered (see figure 9). Upon each answer completion by the participant, the avatar produced a response to the participant’s answer (e.g. ‘Oh, how interesting!’). After having finished the conversation with the third avatar, the experiment was over, and participants were asked to complete questionnaires before they were debriefed on the purpose of the experiment. The study was approved by the Social Sciences Faculty Ethics Committee, Radboud University Nijmegen and informed consent was obtained before and after the experiment.

### 7.5. Behaviour analysis

#### 7.5.1. Answer length

Answer length was measured in seconds (in ELAN 4.9.3, 71) from the first to the last vocalization produced by the speaker in response to each question.

#### 7.5.2. Hesitations

To differentiate changes in answer length owing to content from changes in answer length owing to hesitations, we measured different types of hesitations, namely the frequency and average duration of filled (the Dutch equivalents of *uhs* and *ums* [114]) and unfilled pauses [114,115].

### 7.6. Statistical analysis

We used R (v.3.4.3., [116]) and *lme4* (v.1.1-13) [117] to test in a linear-mixed-effects model whether answer length differed depending on addressee feedback. The initial model was an intercept-only model, estimating the mean answer length including intercepts for items (question stimuli) and participants as random effects (more complex models including random slopes for participants did not converge). Using a likelihood ratio test (using the ‘anova’ function), the intercept model was compared to a model which differed only in that addressee feedback (nod, nod/eyebrow furrow, nod/non-response) was included as a fixed effect. To test whether any effect of addressee feedback on answer length was modulated by the speakers’ empathy, we first entered addressee feedback (nod, nod/eyebrow furrow and nod/non-response) and speaker empathy (EQ score as a scaled and centred continuous variable) as fixed effects (without interaction term) and intercepts for items (question stimuli) and participants as random effects into the model. This model was then compared to a model that only differed in that addressee feedback and speaker empathy were entered as fixed effects *with* interaction term, again using a likelihood ratio test (with the ‘anova’ function). To test whether any effect of addressee feedback on answer length was modulated by the speakers’ FoNE, we first entered addressee feedback (nod, nod/eyebrow furrow and nod/non-response) and FoNE (FoNE score as a scaled and centred continuous variable) as fixed effects (without interaction term) and intercepts for items (question stimuli) and participants as random effects into the model. This model was then compared to a model that only differed in that addressee feedback, and FoNE were entered as fixed effects *with* interaction term, again using a likelihood ratio test (with the ‘anova’ function). To test whether any differences in answer length could be explained by differences in hesitations, we subtracted all filled and unfilled pauses—that is, the sum of durations of all filled and unfilled pauses produced within each answer—from the total length of each answer. Then, we ran the same model comparisons again, as described above, with the only difference that the dependent variable now was ‘answer length minus hesitations’.

## 8. Results: study 2

### 8.1. Speakers’ answer length

**Figure 10.**
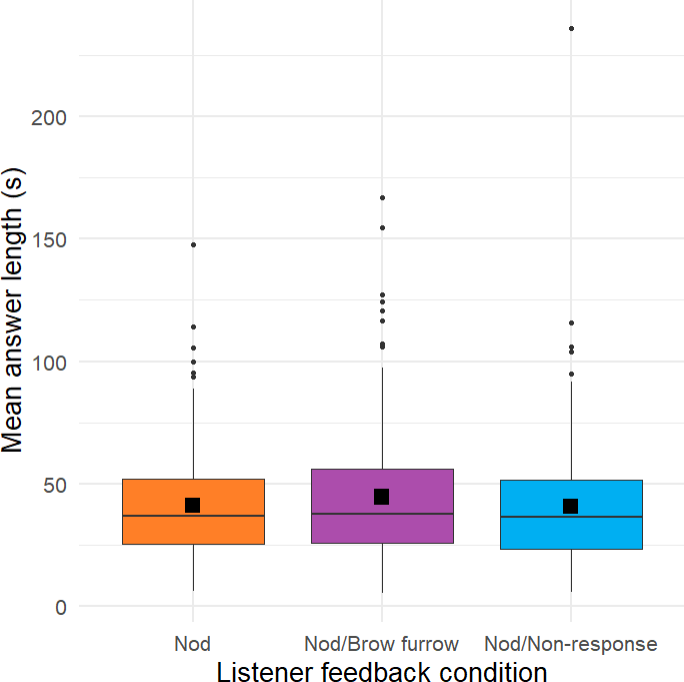
Mean answer length (s) by addressee feedback. Black squares indicate mean per feedback condition. Boxplots indicate median (horizontal line), 25th–75th percentile (box) and ±1.5 interquartile range (whiskers).

Did speakers’ answer length differ depending on addressee feedback? Speakers indeed produced longer answers in the nod/brow furrow condition than in the nod condition. Answers in the control nod/non-response condition were not longer than in the nod condition, as expected (figure 10).^3^

Including ‘addressee feedback’ as fixed effect in the mixed-effect model led to a significantly better fit (*χ*^2^(2) = 6.03, *p* = 0.048), revealing that—relative to avatars that only nodded (41.4 s ± 3.62 (s.e.))—the presence of addressees’ eyebrow furrows increased speakers’ answer length by about 3.77 s ± 1.67 s.e. (*t* = 2.25, *p* = 0.025), that is, by approximately 8–11 words (based on an average of two to three words produced per second in conversation [118]). Also relative to speakers’ answer length in the nod/non-response control condition (41.91 s ± 3.61 (s.e.)), speakers’ answer length was significantly longer in the nod/eyebrow furrow condition (*β* = 3.34, s.e. = 1.68, *t* = 1.98, *p* = 0.047).

Speakers’ answer length in the nod condition and the nod/non-response control condition was statistically indistinguishable (*β* = 0.43, s.e. = 1.68, *t* = 0.25, *p* = 0.798), suggesting that it was not the relatively reduced number of nods in the nod/eyebrow furrow condition that increased the answer length but, as predicted, the presence of eyebrow furrows. Overall, these results support the hypothesis that addressee brow furrows can signal ‘I’ve not received sufficient information for current purposes’, such that speakers provide more information, overall resulting in longer answers.

However, rather than providing additional information, speakers may have produced more hesitations (unfilled, silent pauses and filled pauses like *uh* and *uhm*) when facing an avatar who occasionally furrowed her brows, which may explain the overall longer answers in the brow furrow condition, compared to the nod condition. To address this issue, we subtracted all filled and unfilled pauses—that is, the sum of durations of all filled and unfilled pauses produced within each answer—from the total length of each answer and then tested again in a linear-mixed-effects model whether answer length, now disregarding all filled and unfilled pauses, differed depending on addressee feedback. Again, including ‘addressee feedback’ as fixed effect provided a model with a significantly better fit (*χ*^2^(2) = 9.38, *p* = 0.009), revealing that, relative to avatars that only nodded (27.32 s ± 2.5 (s.e.)), the presence of addressees’ eyebrow furrows increased speakers’ answer length by about 3.26 s ± 1.17 s.e. (*t* = 2.77, *p* = 0.005). Also relative to speakers’ answer length in the nod/non-response control condition (27.57 s ± 2.50 (s.e.)), speakers’ answer length was significantly longer in the nod/eyebrow furrow condition (*β* = 3.00, s.e. = 1.18, *t* = 2.54, *p* = 0.011). Again, speakers’ answer length in the nod condition and the nod/non-response control condition were statistically indistinguishable (*β* = 0.25, s.e. = 1.18, *t* = 0.21). These results indicate that the observed differences in answer length cannot be explained by differences in hesitations, suggesting that, rather than hesitating more, speakers indeed spoke for longer (providing more information content) when facing an avatar who occasionally furrowed her brows compared to an avatar who nodded throughout.

### 8.2. Speakers’ answer length and individual differences in empathy and fear of negative evaluation

**Figure 11.**
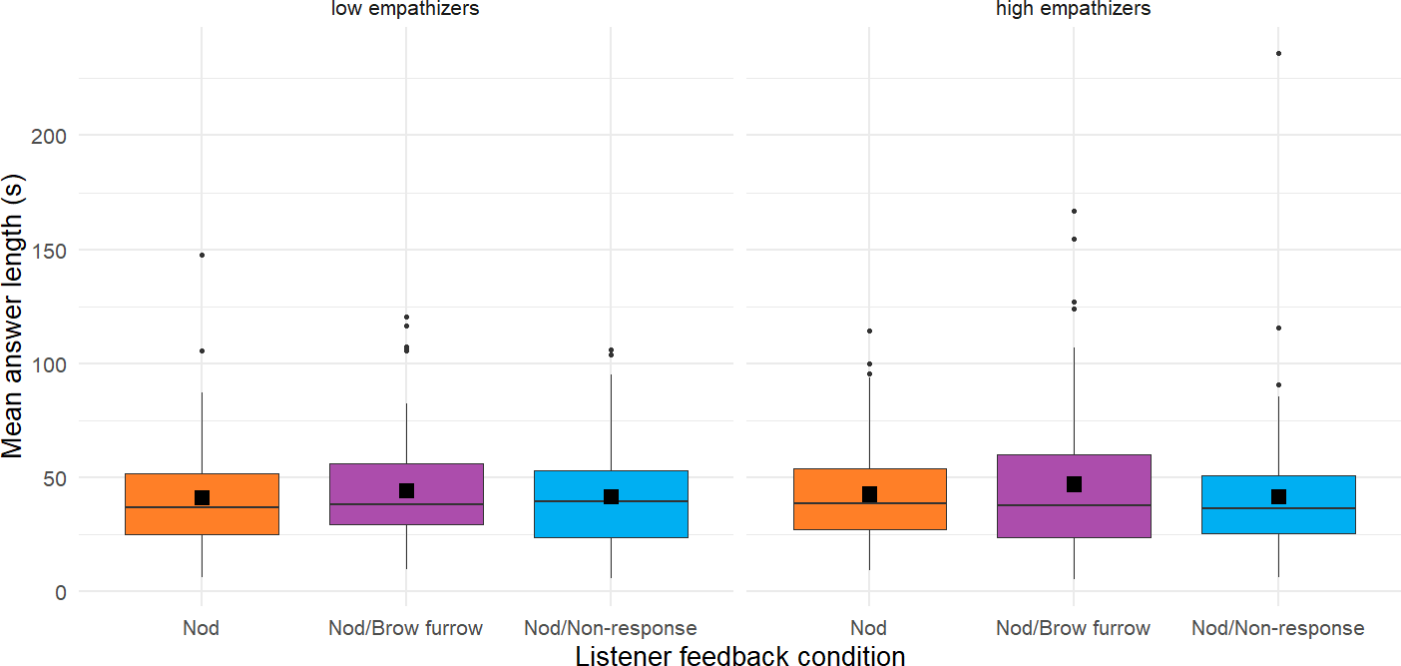
Mean answer length (s) by addressee feedback in low-empathy and high-empathy speakers (median split). Black squares indicate mean per feedback condition. Boxplots indicate median (horizontal line), 25th– 75th percentile (box) and ±1.5 interquartile range (whiskers).

Did the relationship between addressee feedback and speakers’ answer length depend on speakers’ empathy quotient? As figure 11 shows, high-empathy speakers and low-empathy speakers showed similar patterns of results.

We used a linear mixed-effects model to statistically test whether answer length by addressee feedback condition differed depending on the speakers’ degree of empathy, with addressee feedback condition and EQ score as fixed effects (without interaction term). This model was compared to a model that only differed in that addressee feedback and speaker empathy were entered as fixed effects *with* interaction term. Including addressee feedback and speaker empathy *with* interaction term did not provide a model with a significantly better fit (*χ*^2^(2) = 1.77, *p* = 0.40), revealing that the effect of addressee feedback on speakers’ answer length was unaffected by speakers’ degree of empathy, also when disregarding filled and unfilled pauses, that is, when predicting ‘answer length minus hesitations’ (*χ*^2^(2) = 1.77, *p* = 0.32).

**Figure 12.**
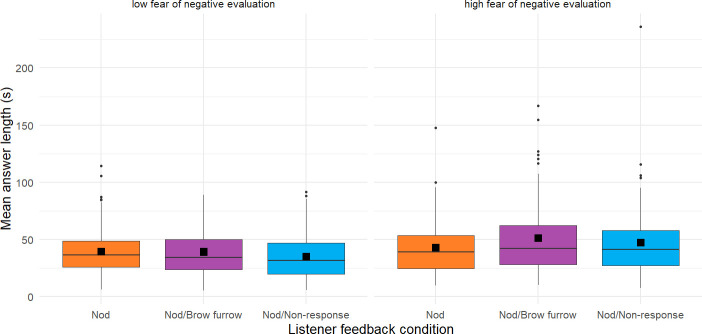
Mean answer length (s) by addressee feedback in speakers with high FoNE versus speakers with low FoNE (median split). Black squares indicate mean per feedback condition. Boxplots indicate median (horizontal line), 25th– 75th percentile (box) and ±1.5 interquartile range (whiskers).

Did the relationship between addressee feedback and speakers’ answer length depend on speakers’ degree of FoNE (figure 12)? To test this, addressee feedback condition and FoNE were entered into a mixed-effects model as fixed effects (without interaction term). This model was compared to a model that only differed in that addressee feedback, and FoNE was entered as fixed effects *with* interaction term. Including addressee feedback and speaker FoNE *with* interaction term improved the model fit only marginally (*χ*^2^(2) = 5.64, *p* = 0.058), revealing that the effect of addressee feedback on speakers’ answer length was not reliably modulated by the speakers’ degree of FoNE, and the same result was obtained when predicting ‘answer length minus hesitations’ (i.e. excluding filled and unfilled pauses from the answer length measure) (*χ*^2^(2) = 3.4, *p* = 0.18). Note that, to explore whether speakers adjusted or marked their speech more locally in response to an addressee’s brow furrow, we also looked at a range of additional variables (speech rate, intensity, pitch change and hesitations), but that none of them explained a significant amount of the data variance (see the electronic supplementary material for these additional analyses).

## 9. Discussion: study 2

The central question study 2 aimed to answer was: are speakers sensitive to addressee eyebrow furrowing as a communicative signal of insufficient understanding? The findings suggest that they are. In this study, speakers produced longer answers when talking to a brow-furrowing addressee than when talking to an addressee that only nodded, thus supporting the hypothesis that addressee eyebrow furrowing can indeed signal insufficient understanding. The observed differences in answer length could neither be explained by differences in hesitations, nor by differences in speakers’ perception of how human or ‘natural’ the virtual addressees appeared as conversational partners.

We were also able to rule out one additional possible alternative explanation. Remember that in the nod/brow furrow condition, a nod was occasionally replaced with a brow furrow, meaning the two conditions did not only differ in the absence versus presence of brow furrows but also in the overall number of nods. Since nods signal understanding, the relatively reduced overall number of nods in the nod/brow furrow condition rather than the presence of brow furrows could have caused speakers to design longer answers than in the baseline nod condition. However, answer length in the control nod/non-response condition did not differ from answer length in the baseline nod condition. This suggests that the difference in answer length between the nod/brow furrow condition and the nod condition cannot be explained by the reduced number of nods but indeed, as hypothesized, by the presence of eyebrow furrows.

## 10. General discussion

Do eyebrow movements play a functional role in signalling communicative problems in spoken face-to-face interaction? This article presents converging correlational and experimental evidence suggesting that they do indeed. In study 1, we have shown that (i) in addition to the linguistic format of the verbal repair initiation, the type of co-occurring eyebrow movement was reliably associated with differences in the linguistic repair format (primarily brow furrows with restricted requests), and (ii) that the type of brow action independently from the linguistic format predicted the type of repair solution. A small number of observations also suggest that eyebrow movements alone may sometimes be sufficient to occasion clarification.

In study 2, we followed up on our corpus-based correlational findings and showed experimentally that speakers are indeed sensitive to addressee eyebrow furrowing as a communicative signal of insufficient understanding (as evidenced by speakers’ longer answers when talking to a brow-furrowing addressee than when talking to an addressee that only nodded), thus supporting the hypothesis that addressee eyebrow furrowing is indeed interpreted as a signal of insufficient understanding.

Our findings have clear theoretical implications. We show that addressee’s facial behaviour can shape the speaker’s speaking behaviour, probably reflecting speaker adjustments at the ‘message level’ [119]. As such, it provides further support for bilateral accounts of speaking, according to which the addressee is an active collaborator coordinating with the speaker moment by moment to maintain mutual understanding [62]. It highlights that speakers in face-to-face communication appear to not only rely on auditory self-monitoring (e.g. [120]) but also on visual other-monitoring (see also [121]). Although natural human language is multimodal and social-interactive in nature, traditional models of language processing have primarily focused on verbal language and on utterances produced outside of a social-interactive context. The studies presented in this article embrace the multimodal as well as the social-interactive, bilateral nature of language, and it provides further motivation for a paradigm shift, an ‘interactive turn’ [122, p. 7] that is already taking place in psycholinguistics [61,123,124], but also in the cognitive sciences more generally [125–127].

While we are suggesting that eyebrow movements serve a communicative function, this does not necessarily entail that they are communicatively intended [128], and future experimental work is required to provide conclusive insights into the extent to which addressee brow furrowing is indeed a communicatively intended, conventional signal. Note, for example, that furrowing the brows might merely be a symptom of the addressees’ processing difficulty or high cognitive load, which is then interpreted and treated by the speaker as indicating a need for clarification. Darwin [7] already mentioned that eyebrow furrows (or ‘frowns’, as he called them) are not only associated with unpleasantness but also with a potentially related but distinct state of dealing with difficulty in thought:

‘A man may be absorbed in the deepest thought, and his brow will remain smooth until he encounters some obstacle in his train of reasoning, or is interrupted by some disturbance, and then a frown passes like a shadow of his brow*’* [7, p. 221]

**Figure 13.**
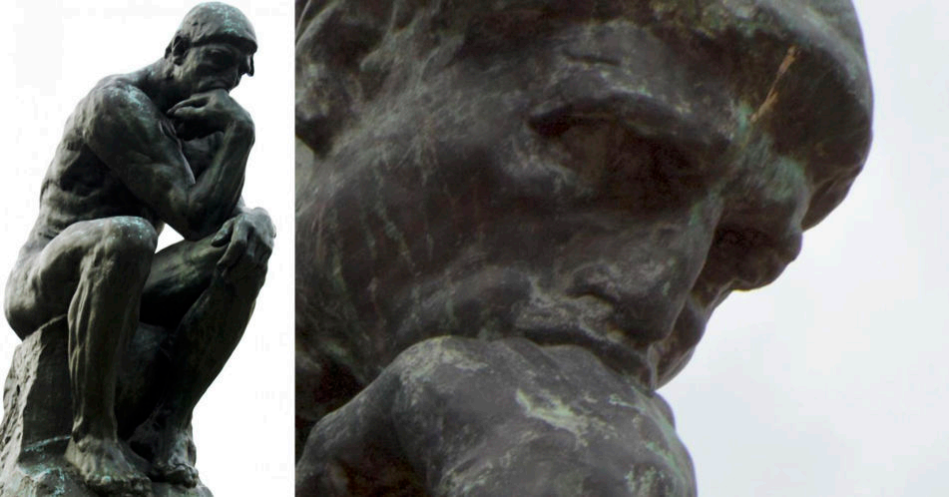
Rodin’s sculpture *Le Penseur* (‘The Thinker’, 1880) and a facial close-up showing his furrowed eyebrows. Note that the philosopher Gilbert Ryle famously used *Le Penseur* in the mind-body debate, asking ‘What is he doing?’ [129], arguing against the privacy of cognitive states. Photo on the left: retrieved from https://pxhere.com/en/photo/1127793, CCO. Photo on the right: retrieved from https://www.flickr.com/photos/japanexperterna/16543319711/, CC BY-SA 2.0, cropped.

The observation that people—as individuals not engaged in conversation—also furrow their brows when dealing with cognitive difficulties suggests that such furrows may not only serve other-oriented, communicative function in signalling a need for clarification in conversation but that they may also serve a self-oriented, cognitive function (see figure 13 for an illustration).

Social-communicative functions and potential cognitive, perceptual and emotional functions of eyebrow movements are not mutually exclusive. It is possible that the cognitive, perceptual and emotional functions underlie and precede the communicative signalling function, phylogenetically as well as ontogenetically (e.g. [130] reports eyebrow furrows during ‘concentration’ already in one to three-month-old infants). The eyebrow furrow as a potential symptom of mental effort, for example, may have been co-opted for communicative purposes through processes of ritualization [7,23,131–133], which would point to a non-arbitrary, iconic relationship [134,135] between form and function in communicative eyebrow furrows. In the same way in which closing the eyes by blinking may signal ‘no need to see anymore’ because sufficient understanding has been reached [65,95], furrowing the eyebrows—as if trying to see more clearly^4^,^5^—appears to signal insufficient understanding, potentially shedding new light on the suggested ‘embodied’ origin of the ‘understanding-is-seeing’ metaphor [137] and on visual origins of mental-state signalling [59].

Our results suggesting a communicative function of eyebrow movements in signalling informational needs in spoken Dutch are in line with studies on eyebrow movements in Dutch sign language [27,138] and Argentine sign language [30,55]. This suggests that eyebrow movements as signals of insufficient hearing or understanding may be independent of language modality—since they are used in spoken as well as signed language—as well as from language history—since they have been described in unrelated languages. If the use of eyebrow movements as a signal of insufficient hearing or understanding is stable across a variety of unrelated languages, this would be consistent with Darwin [7, p. 221] who noted ‘the Australians, Malays, Hindoos, and Kafirs of South Africa frown, when they are puzzled’ and who suggested that ‘men of all races frown when they are in any way perplexed in thought’, but it may also suggest that eyebrow movements as signals of communicative problems have evolved from common pressures of a shared conversational infrastructure [139–143].

### 10.1. Limitations and future directions

The present study also has a number of limitations. One is that the present data does not include eye-tracking measurements, nor any measurements of peripheral visual perception, making it difficult to know with certainty whether participants perceived individual addressee eyebrow movements or not. Cases where they did not may have added noise to the data, meaning the current findings may underestimate the effect eyebrow movements associated with conversational repair can have in interaction. Furthermore, the pseudo-interactions between participants and the avatars from study 2 remain a rough approximation of human–human interactions and may differ from them in several ways. However, past research in the language and cognitive sciences has replicated core behaviour patterns defining human performance in experiments employing avatars, such as syntactic priming, speech rate accommodation or non-verbal mimicry [113,144,145]. This suggests that the behavioural findings from the present study may generalize to human–human interaction, especially in conjunction with the conversational next turn-analyses the avatar experiment was based on. Nevertheless, an interesting avenue for future studies will be investigations into the extent to which increased realism (especially photorealistic avatars), fully interactive and immersive (three-dimensional) settings may be of relevance for capturing the effects of visual signals in conversational human–artificial agent interaction, including eyebrows and repair. This also includes the more systematic investigation of empathy and other social and personality traits that may modulate face-to-face interaction and multimodal repair processes in human–avatar interaction. A third limiting factor is the confederate design applied in study 2. While we made sure that the confederate was blind to the experimental hypotheses, had full access to the visual and verbal behaviour of the participant, was free to respond to them whenever they deemed it appropriate and the number of button presses was comparable across conditions, it is of course possible that the artificial manner of responding (button press) and the repeated exposure to the experimental set-up may have led to addressee feedback behaviour that was perceived as unnatural in timing or form (i.e. the way in which it was rendered on the avatar). The advantages but also potential drawbacks of confederate designs have been detailed in the literature [146,147], making future replications with non-confederates an important next step. Finally, the statistical power in some of our analyses was on the lower side, meaning that some of the results that were not statistically significant may emerge as such in future comparisons.

To conclude, the results suggest that—in addition to visual, emotional and possible cognitive functions—eyebrow furrowing may serve as a cooperative signal of insufficient understanding and may facilitate achieving mutual understanding in face-to-face conversation by influencing how interlocutors respond to signals of trouble in understanding (in terms of the amount and type of information provided). As such, the present findings contribute to our understanding of how participants in human face-to-face interaction coordinate.

## Data Availability

The video data from Study 1 and 2 cannot be shared for ethical restriction reasons, but the raw, quantitative data based on the video data, which served as the basis for the analyses reported, has been stored on OSF and is accessible via a view-only link: https://osf.io/rnv9z/?view_only=cf50164be0494c91b9a7c8dc031b6ad6. Supplementary material is available online [148].

## Footnotes

^1^
Type of eyebrow action excluded verbal-only cases, resulting in 268 observations from 83 speakers in 55 dyads.

^2^
Out of the 95 OIR cases in which the repair solution was absent, 41 were verbal-only repair initiations, 32 with a brow furrow and 22 with a brow raise.

^3^
Predicting the confederate’s feedback button press frequency (number of button presses per answer divided by the length of the same answer in minutes; *M* = 10.74; s.d. = 3.52) by feedback condition (nod, nod/brow furrow and nod/non-response), including random intercepts for participants and items, confirmed that button press frequency was consistent across conditions (nod versus nod/brow furrow: *β* = −0.05*,* s.e. = 0.25, *t =* −0.255*, p* = 0.822; nod versus nod/non-response: *β* = 0.231*,* s.e. = 0.253, *t =* 0.913*, p* = 0.362; nod/brow furrow versus nod/non-response: *β* = 0.288*,* s.e. = 0.253, *t =* 1.138*, p* = 0.256).

^4^
According to Darwin [7, p. 221], Prof. Donders already suggested that eyebrows are furrowed to see more clearly (‘the corrugators are brought into action in causing the eyeball to advance in accommodation for proximity in vision’).

^5^
See also [136] on the ‘thinking face’, referring to the speaker marking a word search by turning away her gaze from the addressee with a distant look and with a facial gesture of someone thinking hard.
